# Investigating the Effects of Intrinsic Motivation and Emotional Appeals Into the Link Between Organic Appeals Advertisement and Purchase Intention Toward Organic Milk

**DOI:** 10.3389/fpsyg.2021.679611

**Published:** 2021-10-04

**Authors:** Jianming Wang, Wilson Dang, Wang Hui, Zheng Muqiang, Wu Qi

**Affiliations:** ^1^School of Business Administration, Zhejiang University of Finance & Economics, Hangzhou, China; ^2^Department of Business Administration, Dong Nai Technology University, Bien Hoa, Vietnam; ^3^Business School, Xiangtan University, Hunan, China; ^4^Department of Applied Economics, Business School, Shantou University, Guangdong, China

**Keywords:** Organic advertisement, organic milk, purchase intention, intrinsic motivation, emotional appeals

## Abstract

Consumers care about healthy food. Thus, several firms use organic appeals advertising to change consumer attitudes and persuade them to purchase organic food. Organic appeals advertisement often presents content that provides information and knowledge about organic elements of a food product (e.g., health, safety, a lack of chemicals, and rich nutrition). In contrast, non-organic appeals advertisement does not present information about organic elements of a food product. This study aims to clarify the effect of organic appeals advertisement on consumer motivations and behavior. It uses the stimuli-organism-response model and self-determination motivation theory to investigate the relationship between organic appeals advertisement and purchase intention toward organic milk considering the mediating role of intrinsic motivation and the moderating role of emotional appeals. Two experimental designs are used to test the hypotheses. Results show that consumers receiving organic appeals advertisement have a higher intention to purchase organic milk than those receiving non-organic appeals advertisement. Furthermore, intrinsic motivation is found to have a mediating role in the link between organic appeals advertisement and purchase intention. In other words, when consumers receive advertisements of an organic milk product, they have higher intrinsic motivation and hold higher intention to purchase organic milk products. Furthermore, emotional appeals have a moderating effect on the relationship between organic appeals advertisement and intrinsic motivation. The influence of organic appeals advertisement on intrinsic motivation is stronger when emotional appeals are positive and weaker when emotional appeals are negative.

## Introduction

Organic food consumption has been a research focus in recent decades (Boobalan et al., 2021; van de Grint et al., 2021). With increased income and consumption knowledge, consumers are demanding more green products (Molinillo et al., 2020). Organic food is among the most demanded type of green product in today’s environment. The reason is that consumers often view organic food as safer and healthier than conventional food (Suciu et al., 2019). However, organic food consumption remains low compared with conventional food because consumers lack useful information and knowledge and are unmotivated to purchase organic food (Sultan et al., 2020). Given the benefits of organic food to human health, researchers and business managers should understand factors that lead to consumers’ motivation and purchase behavior toward organic food (Molinillo et al., 2020).

Organic milk is one of the most frequently consumed products (Carfora et al., 2019). Organic milk refers to several types of milk products from livestock raised according to organic farming methods. Organic milk is produced and processed without chemicals or any ingredients that harm human health (Barański et al., 2017). More and more people purchase organic milk to replace conventional milk (Scozzafava et al., 2020). However, consumption of organic milk is still largely lower than conventional milk (Carfora et al., 2019). Scozzafava et al. (2020) explained that a lack of helpful information and knowledge about organic milk is the main factor that affects consumer preferences and willingness to pay for organic milk. Thus, the authors called for more research to determine the consumption of organic milk.

Advertising is among several effective marketing strategies used to influence and persuade consumers to purchase products and services (Kotler and Armstrong, 2018). In some situations, marketers use advertising to inform and educate consumers to understand a particular product (Kim et al., 2020b). In organic milk, consumers may lack helpful information and knowledge to distinguish between organic and conventional milk. Organic appeals advertising may provide advanced information and knowledge that help consumers understand the benefits of organic milk (Rauwers et al., 2018). Organic appeals advertising refers to message content and information that explain and emphasize organic elements of a product, such as milk (Yang and Ghose, 2010). In particular, organic appeals advertising often indicates that organic milk is naturally produced with rich nutrition, is processed without chemicals, and is healthy and safe for consumers (Anghelcev et al., 2020). By contrast, non-organic appeals advertising does not present and emphasize organic elements of a milk product (Carfora et al., 2019). Organic appeals advertising may be an effective way to provide useful information and knowledge for consumers to understand organic milk products better (Anghelcev et al., 2020). Although several firms have widely used organic appeals advertising to educate and persuade consumers (Wu et al., 2019), the effect of organic appeals advertising on consumers’ motivation and behavior has been unclear and lacks empirical evidence in prior literature. This research gap needs to be addressed in the current study.

Furthermore, consumers are often motivated by internal or external factors to purchase certain products (Soroka and Wojciechowska-Solis, 2019). When consumers receive a signal from the external environment, they may be internally motivated and take action toward purchasing a particular product because they find purchasing the product with their internal needs necessary (Standage et al., 2006). In organic milk, when consumers receive information from organic appeals advertisements, they understand the unique benefits and the difference between organic milk and conventional milk. Therefore, organic appeals advertisement may trigger consumers’ internal motivation, leading to consumers’ behavioral intention toward organic milk. In other words, intrinsic motivation may play a mediating role in the link between organic appeals advertisement and purchase intention toward organic milk. Unfortunately, the mediating mechanism of intrinsic motivation in affecting consumers’ purchase behavior of organic milk has been unexplored in prior literature.

Furthermore, emotion is an important factor that influences a person’s perceptions, attitudes, and behavioral outcomes (Solomon, 2018). Emotion also affects consumer decision-making (Lajante and Ladhari, 2019). Consumers who experience a negative emotion are more likely to have bad feelings and negative attitudes, leading to negative behavior (Harrison-Walker, 2019). By contrast, positive emotion makes consumers feel comfortable and pleasant and encourages consumers to shop (Septianto and Chiew, 2018). Given the importance of emotion, positive emotional appeals may increase the effectiveness of organic appeals advertising and enhance consumers’ motivation toward organic milk because positive emotion often leads to consumer positive perceptions, attitudes, and behavior (Ng and Diener, 2009). However, negative emotional appeals may reduce the influence of organic appeals advertising on consumers’ motivation because bad feelings and negative emotions prevent consumers from enjoying shopping (Song and Qu, 2019). The role of emotions in affecting consumers’ attitudes and behavior in the shopping process is important. However, the moderating role of emotional appeals on the link between organic appeals advertising and consumers’ intrinsic motivation toward organic milk has been largely ignored in prior literature.

This study fills these gaps by investigating the relationship between organic appeals advertisement and purchase intention toward organic milk, with the mediating role of intrinsic motivation and the moderating role of emotional appeals. This study contributes to the current literature in three ways. First, this study extends stimuli-organism-response (SOR) theory into the field of organic food research and uses it as a theoretical foundation to explain the direct influence of organic appeals advertisement on purchase intention toward organic milk. Second, based on self-determination motivation (SDT) theory, this study demonstrates that organic appeals advertisement increases consumers’ intrinsic motivation, enhancing consumers’ intention to purchase organic milk. This mediating mechanism of consumers’ intrinsic motivation helps to clarify the indirect effect of organic appeals advertisement on consumer purchase intention. Thus, this study sheds new light on the direct and indirect influence of organic appeals advertisement on purchase intention toward organic milk. Third, this study shows that positive and negative emotional appeals have different effects on the relationship between organic appeals advertisement and consumers’ intrinsic motivation. This moderating mechanism of emotional appeals advances our knowledge to understand how emotional appeals influence consumer behavior in the consumption of organic milk.

## Theories and Hypotheses

### SOR Theory

stimuli-organism-response has been widely applied in psychology and consumer behavior fields (Wu and Li, 2018). SOR is used to explain the relationship between environmental stimuli (S), organism (O), and behavioral response (R; Mehrabian and Russell, 1974). Specifically, environmental stimuli influence a person, eliciting a response (Kim et al., 2018). In consumer behavior research, external factors in the environment are considered as stimuli (S; e.g., advertising, brand, product, and price), the internal process when consumers receive the influence of external factors is considered the organism (O; e.g., perception, memory, recognition process), and consumers taking action toward external stimuli is considered the response (R; e.g., purchase behavior and word of mouth; Peng and Kim, 2014).

Several studies have used SOR theory to explain consumer behavior toward the consumption of food in prior literature (Kim et al., 2020a). For example, Talwar et al. (2021) adopted SOR theory. They found that food safety concerns and health consciousness positively influence openness to change and ethical self-identity, increasing consumers’ willingness to buy organic food. Shah et al. (2021) used SOR and clarified the impact of different components of mobile dining on customers’ perceived value, which leads to actual purchase intentions. Liang and Lim (2021) adopted the SOR model to create a comprehensive model to explain consumers’ purchase decisions toward organic food. The authors found that consumer preference for natural food was the most important factor for enhancing purchase intention, followed by health consciousness, health risk, attitude toward organic food, and trust in labeling. Liu and Zheng (2019) based their study on SOR theory. They explained that consumer characteristics, food safety incidents, environmental orientation, and consumer health orientation positively relate to consumer organic cognition and purchase behavior. Given the importance of SOR in explaining the relationship between external factors and consumer responses in prior literature, this study applies SOR theory to explain the influence of organic appeals advertising on purchase intention toward organic milk.

### Organic Appeals Advertisement and Purchase Intention

Advertising is a powerful promotional tool used by firms to influence consumers’ perceptions, attitudes, and behavior (Kotler and Armstrong, 2018). Specifically, marketers often use advertising to change consumers’ attitudes and persuade them to purchase products and services (Shaouf et al., 2016). In general, advertising appeals can be divided into emotional and rational appeals. The former refers to advertising that can elicit consumers’ negative or positive emotions (e.g., adverts that are funny, lovely, sad, or charming). In contrast, the latter refers to advertising that indicates the benefits and values of products and services for consumers (Kotler and Armstrong, 2018). Belch and Belch (2004) suggested that rational advertising focuses on consumers’ real needs toward a product by emphasizing the characteristics, values, and benefits consumers would have if they bought and used a product. Rational advertising is often used to inform and persuade that the new product is superior to existing products (Kotler and Armstrong, 2018).

In the last few years, the demand for organic milk products has been gradually increasing. The reason is that consumers demand more healthy and safe milk products (Boobalan et al., 2021). However, although organic milk is superior to traditional milk in several ways, a lack of useful information and knowledge has prevented consumers from purchasing organic milk (Scozzafava et al., 2020). Several firms have used organic appeals advertising to educate and persuade consumers (Rauwers et al., 2018). However, the effect of organic appeals advertising on consumer purchase behavior has been untested and unclear in prior literature, limiting our understanding of the different effects between organic and non-organic appeals advertising. Thus, the low consumption of organic milk products must be because consumers care about their health and safety (Carfora et al., 2019; Scozzafava et al., 2020). According to SOR theory (Mehrabian and Russell, 1974), organic appeals advertising can be viewed as an external stimuli (S) that exerts an influence on consumers (O), who will take action (R) toward external stimuli. Specifically, when exposed to organic appeals advertising, consumers may notice and understand the benefits of organic milk. As a result, they may hold a high intention to purchase organic milk because they believe that consumption of organic milk brings health and hygiene (Qin et al., 2009). In other words, organic appeals advertising provides valuable information and knowledge about the benefits of organic milk for consumers. In this case, organic appeals advertising provides information to remind consumers, educates, and offers knowledge for consumers to understand and distinguish between organic and non-organic milk products. Given the information and knowledge received from advertising appeals, consumers may understand the superiority of organic milk compared with conventional milk. As a result, consumers become more likely to purchase organic milk. The reason is that consumers increasingly care about their health and safety while having enough information and rich knowledge about organic milk products obtained from advertisements (Jaeger and Weber, 2020). By contrast, when consumers watch non-organic appeals advertising, they may not fully understand the superior benefits of organic milk and the difference between organic and non-organic milk products. A lack of knowledge and information about organic milk may reduce consumers’ willingness to buy organic milk because consumers cannot distinguish between organic and conventional milk (Bloksma et al., 2008). In this case, buying organic milk is not attractive to consumers because they may believe that organic milk is not likely to differ from conventional milk. Therefore, the following hypothesis is developed.

*H1*: Consumers receiving organic appeals advertising have greater purchase intention toward organic milk than those receiving non-organic appeals advertising.

### SDT Theory

self-determination motivation is often widely used in psychology, organizational behavior, and education (Stupnisky et al., 2018). SDT refers to an individual’s motivation to accomplish a specific objective. That is, motivation acts as a driving mechanism to guide an individual’s behavior toward an end goal (Ryan and Deci, 2000). In SDT, two types of motivation are distinguished: extrinsic and intrinsic motivation (Welters et al., 2014). Extrinsic motivation indicates that an individual’s behavior is motivated by external or instrumental reasons (e.g., rewards and punishments). In contrast, intrinsic motivation occurs when an individual engages in an activity for the enjoyment inherent in its activity (Howard et al., 2016).

self-determination motivation has been widely used to explain consumers’ motivation and behavior in organic food literature. For example, Dang et al. (2021) used SDT. They found that perceived healthiness and environmental consciousness are positively related to extrinsic motivation, which positively influences purchase intention toward organic drinking products. Shamsi et al. (2020) demonstrated the predictive ability of SDT on organic food consumption behavior. Tando et al. (2020) showed a significant influence of intrinsic motivation and integrated and external regulation on consumer attitude and buying behavior of organic food. Chiu et al. (2019) found that self-determination has a positive influence on personal relevance, which positively affects customer citizenship behavior toward organic food. Schösler et al. (2014) indicated that internalized motivation is the main factor that makes a difference in the intrinsic enjoyment of cooking and eating behavior. Prior studies have provided rich evidence for the predictive ability of SDT on consumers’ behavioral outcomes. Based on the evidence of SDT literature, this study examines the mediating role of intrinsic motivation in the relationship between organic appeals advertisement and purchase intention toward organic milk.

### Mediating Role of Intrinsic Motivation

Intrinsic motivation often refers to individuals’ internal motives that drive and guide their actions toward an objective (Ryan and Deci, 2000). When individuals receive a signal from external stimuli, their internal motivation may be triggered through a cognitive process, in which they find performing a particular task necessary. This action occurs given the willingness and inner pleasure that motivates individuals to engage voluntarily (Gagné and Deci, 2005). For example, a person watching a sports video may find engaging in sport activities necessary. This person may feel pleasant and enjoy his sports activities because of internal motivation and not because of external rewards or punishment (Standage et al., 2006).

In the case of organic milk, organic appeals advertisement may act as an external clue that provides a signal for consumers. When consumers watch an organic appeals advertisement, they receive valuable information and knowledge about organic milk products (Xu et al., 2012). Given their understanding of organic milk, they may be triggered by their internal needs because they view organic milk as healthy products that provide more nutrition and benefits for human health (Suciu et al., 2019). Consequently, internal motivation may guide consumers’ attitudes and behavior toward organic milk products because consumers may consume organic products necessary for their health and wellbeing (Kushwah et al., 2019). For example, people often enjoy a particular food because they find it delicious. Sometimes, people also enjoy the food because it is healthier and safer than other food. One specific instance is the case of McDonald’s and Subway. Many consumers enjoy McDonald’s food because they feel McDonald’s food is delicious. However, many other consumers may internally enjoy Subway’s food because it is tasty, organic, and healthy. In this case, consumers internally want Subway’s food because they know that Subway’s food is organic and healthy (Kotler and Armstrong, 2018). Therefore, according to SDT, consumers are internally motivated to receive helpful information and knowledge from organic appeals advertising. The reason is that consumers understand the benefits of organic milk and consuming such products necessary. Consequently, consumers tend to hold high intention to purchase organic milk because consuming organic milk is often a voluntary behavior that generates internal enjoyment and pleasure for consumers, given that consumers know the superior benefits of organic milk. In other words, organic appeals advertisement triggers consumers’ intrinsic motivation, which drives consumers’ behavioral intention toward purchasing organic milk. Thus, the following hypothesis is developed.

*H2*: Intrinsic motivation positively mediates the relationship between organic appeals advertisement and purchase intention toward organic milk.

### Moderating Role of Emotional Appeals

Emotion often plays a vital role in people’s daily life because it affects their feelings, attitudes, and behavioral outcomes (Kotler and Armstrong, 2018). Negative emotions may make them feel uncomfortable, get angry, and engage in negative actions. In contrast, positive emotions elicit positive attitudes and feelings that generate positive behavior (Ng and Diener, 2009). Emotion has been a focus of research in psychology (Kobylińska et al., 2020).

In marketing and consumer behavior, emotion is often considered an important factor that affects consumer decision-making (Lajante and Ladhari, 2019). In the case of negative emotional appeals, consumers experience a negative feeling. This emotional state triggers psychological stress and uncomfortable feelings, which prevent consumers from shopping behavior (Xu, 2020). For example, consumers who experience a negative emotion are more likely to complain and discourage engaging in purchasing behavior (Harrison-Walker, 2019). Thus, when experiencing a negative emotion, consumers may reduce their motivation toward a particular product because of their uncomfortable feeling and psychological distress (Song and Qu, 2019).

In contrast, in the case of positive emotion, consumers experience a happy and pleasant feeling. They are encouraged to engage in purchasing behavior because they find that shopping is a hedonic process (Septianto and Chiew, 2018). That is, positive emotion motivates consumers to take active action toward shopping behavior in which consumers can enjoy shopping as a comfortable and entertaining process (Das and Varshneya, 2017).

Consumers’ emotional responses are often the result of emotional appeals within a marketing stimulus (e.g., advertising and music; Solomon, 2018). Emotional appeals can be either negative or positive (Kotler and Armstrong, 2018). A negative emotional appeal can trigger consumers’ negative emotional response, whereas a positive emotional appeal can elicit consumers’ positive emotional response (Ng and Diener, 2009). Given that emotional appeals lead to consumer emotional responses, consumers’ motivation, attitudes, and behavior may be influenced by their emotional state (Solomon, 2018). Furthermore, negative and positive emotional appeals may affect the relationship between organic appeals advertisement and consumers’ intrinsic motivation differently (Song and Qu, 2019).

On the one hand, the influence of organic appeals advertisement on intrinsic motivation may be decreased when consumers experience a negative emotion. The reason is that negative emotion leads to unpleasant feelings, which generate negative attitudes and reduce consumers’ motivation and willingness toward organic milk products (Jin et al., 2020). For example, when a marketer uses negative emotional appeal within a marketing stimulus (e.g., a sad music stimulus) to make consumers experience a negative feeling, consumers may have low motivation to interpret the advertisement and enjoy organic milk products. Negative emotion exerts psychological distress that prevents consumers from watching and interpreting an advertising message and understanding the benefits of a milk product (Solomon, 2018). In this case, negative emotional appeals trigger consumers’ negative emotional responses, which decreases their attention and motivation. That is, negative emotional appeal lessens the relationship between organic appeals advertisement and consumers’ intrinsic motivation toward organic milk products.

On the other hand, the effect of organic appeals advertisement on intrinsic motivation may increase when consumers experience a positive emotion. Positive emotion motivates and encourages consumers because comfortable feelings and pleasant experiences generate positive attitudes, leading to active engagement (Septianto and Chiew, 2018). For example, when a marketer uses a positive emotional appeal within a marketing stimulus (e.g., a happy music stimulus) to make consumers experience positive emotion, consumers may be encouraged to focus more attention and interpret the message content of an organic appeals advertisement. They are also motivated to take positive actions toward the advertised product (Kotler and Armstrong, 2018; Solomon, 2018). In this case, positive emotional appeals elicit consumers’ positive emotional response, which encourages them to direct their attention and efforts to the content of organic appeals advertisement and motivates them toward organic milk products. That is, positive emotional appeal strengthens the influence of organic appeals advertisement on consumers’ intrinsic motivation toward organic milk products. Therefore, emotional appeals (negative and positive emotion) influence the relationship between organic appeals advertisement and intrinsic motivation differently. Thus, the following hypothesis is developed.

*H3*: Emotional appeals moderate the relationship between organic appeals advertisement and intrinsic motivation toward organic milk.

**Figure 1:**
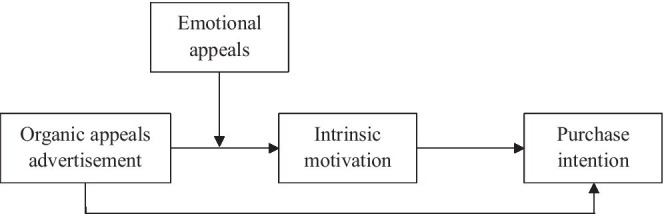
Presents the research model.

Figure 1 shows the research model in this study.

## Methods and Results

### Study 1

#### Pilot Test

We conducted a pilot test using a between-subjects design to check the initial validity of two pieces of advertisement of the same milk brand (organic vs. non-organic appeals advertisements). Fifteen respondents were divided between an organic group with eight participants and a non-organic group with seven participants.

Our research team employed the keywords “organic milk appeals advertising” and “non-organic milk appeals advertising” to find advertisements on Youku and Tencent Video, which are the two largest video channels (similar to YouTube) in China. China only has a few milk companies that have used organic appeals advertising. Only a few advertisements were found. A brand that is the largest milk company in China was selected. Our research team watched all of this brand’s ads streamed in the last three years. We considered each advertisement’s contents and suitability with the purpose of this study. Then, we selected one organic appeals advertisement and another non-organic appeals advertisement. The video of the organic appeals advertisement lasted approximately 1.1min. This advertisement presented several pieces of information about the organic elements of organic milk products (e.g., this milk product is organic. It is produced naturally from an organic farm, processed without any chemical elements, and is healthier and safer than traditional milk products). By contrast, the video of a non-organic appeals advertisement lasted approximately 1.8min. It did not have any information about the organic elements of milk products.

Then, respondents responded to the statement “This advertisement is about an organic milk product” on a seven-point Likert-type scale (1=strongly disagree, 7=strongly agree) to evaluate the two advertisements. The results show that organic appeals advertisement reflects organic milk products and non-organic appeals advertisement reflects non-organic milk products (*t*=7.58, *df*=14, *p*<0.001, *M*_difference_=3.47).

#### Manipulation Check

To check the validity of the pilot test, we performed a manipulation check (between-subjects design) with 44 students enrolled in an undergraduate business course. The respondents were randomly assigned: 18 students were placed in the first group and watched a non-organic appeals advertisement, and 26 students were placed in the second group and watched an organic appeals advertisement. The two advertisements were adopted from the pilot test. After watching the advertisements, the respondents responded to the statement “This advertisement is about an organic milk product” on a five-point scale (1=strongly disagree, 5=strongly agree). The results show a significant difference between organic appeals and non-organic appeals advertisements (*t*=14.53, *df*=43, *p*<0.001, *M*_difference_=3.68). Thus, the manipulation of the independent variable in our experiment was effective.

#### Sample Data

The purpose of study 1 is to test the direct effect of organic appeals advertisement on purchase intention. The experiment was conducted in May 2020 in a large university in China. A total sample of 83 undergraduate students voluntarily participated in the experiment. The sample had 43 women (51.8%) and 40 men (48.2%). These students come from different majors, including art and literature (8 students), business administration (24 students), engineering (20 students), sport & leisure (14 students), computer & information (4 students), and medicine (13 students). The respondents also reported their frequency of drinking milk products: sometimes (8 respondents, 9.6%), usually (46 respondents, 55.4%), daily (25 respondents, 30.1%), and addiction (4 respondents, 4.8%).

#### Ethical Consideration

In this study, experimental design involves human activity. We complied with ethical standards and obtained approval from the Major Project of China’s National Social Science Fund. The respondents were asked to participate in the experiment voluntarily and were provided with anonymity measures.

#### Measures

Purchase intention of organic milk was measured with three items from Prakash et al. (2018): “I am willing to buy organic milk while shopping,” “I will make an effort to buy organic milk in the near future,” and “I intend to buy organic milk.” The Cronbach’s alpha for this measure was 0.89.

#### Analysis and Results

The results of ANOVA show that consumers who watched organic appeals advertisement (*M*_organic_=4.705, *SD*=0.314, *N*=52) held higher purchase intention toward organic milk than those who watched non-organic appeals advertisement (*M*_non-organic_=2.172, *SD*=0.564, *N*=31; *F*=693.231, *df*_between_=1, *df*_within_=81, *df*_total_=82, *p*<0.001). The results of study 1 support hypothesis H1.

### Study 2

#### Pilot Test

We conducted a pilot test using a between-subjects design with 10 respondents (positive emotion group with five participants and negative emotion group with five participants) to check the initial validity of emotional appeals (happy song vs. sad song). We used the keywords “sad songs” and “happy songs” and found different songs on QQ-Music, one of China’s top music apps. From a list of sad songs and a list of happy songs, we selected the saddest song and the happiest song ranked by users in the last month on QQ-Music. After listening to these two songs, we discussed their suitability with the purpose of this study. Then, we decided to use these two songs for our experiment.

The respondents responded to the statement “This song makes me happy” on a seven-point Likert-type scale (1=strongly disagree, 7=strongly agree) to evaluate the two songs. The results ensured that the happy song and the sad song could elicit different emotions (*t*=6.05, *df*=9, *p*<0.001, *M*_difference_=3.40).

#### Manipulation Check

To check the validity of the pilot test, we performed a manipulation check (between-subjects design) with the participation of 49 consumers. The respondents were randomly assigned: 20 consumers were placed in the first group and listened to a sad song, and the other 29 consumers were placed in the second group and listened to a happy song (these two songs were adopted from the pilot test). After listening to the songs, the respondents responded to the item “This song makes me happy” on a five-point scale (1=strongly disagree, 5=strongly agree). The results show a significant difference between the happy song and sad songs groups (*t*=15.31, *df*=48, *p*<0.001, *M*_difference_=3.49). Thus, the manipulation of the mediator variable in our experiment was effective.

#### Sample Data

In Study 2, we invited 170 consumers who shop at different superstores to join our experiment. The experiment was conducted in August 2020 in Guangzhou, China. A total of 155 consumers agreed to participate in the experiment. We announced to the respondents that their participation in this study was purely voluntary and assured them of the anonymity of their responses. We offered each respondent 20 USD after the experiment to thank them for their participation. The characteristics of respondents are present in Table 1.

**Table 1:** Respondents’ characteristics.

Variable	Frequency	Percent
Age		
20 and below	24	15.5%
21–30	71	45.8%
31–40	45	29.0%
41 or above	15	9.7%
Gender		
Male	80	51.6%
Female	75	48.4%
Income		
Under 100 USD	9	5.8%
100–under 200 USD	11	7.1%
200–under 300 USD	34	21.9%
300–under 400 USD	37	23.9%
400–under 500 USD	17	11.0%
500 USD or above	47	30.3%
Education		
High school or below	16	10.3%
Undergraduate	135	71.0%
Master or above	29	18.7%

*n*=155.

#### Ethical Consideration

In Study 2, we also complied with ethical standards and obtained approval of the Major Project of China’s National Social Science Fund. Respondents were asked to participate in the experiment voluntarily and were provided with anonymity measures.

#### Experimental Procedure

We conducted a 2 (organic vs. non-organic)×2 (positive vs. negative appeals) between-subjects design. The respondents were randomly assigned into one of the four groups. In Group 1, the respondents watched an organic milk advertisement and then listened to a happy song. In Group 2, the respondents watched a non-organic milk advertisement and then listened to a sad song. In Group 3, the respondents watched an organic milk advertisement and then listened to a sad song. In Group 4, respondents watched a non-organic milk advertisement and then listened to a happy song. After that, all respondents completed a questionnaire that measures their intrinsic motivation and purchase intention toward organic milk.

#### Measures

The mediating variable (i.e., intrinsic motivation) was measured using three items adapted from Lin et al. (2009). These items include “I enjoy the consumption of organic milk,” “consumption of organic milk is attractive,” and “consumption of organic milk is enjoyable.” The Cronbach’s alpha of intrinsic motivation was 0.85. Furthermore, purchase intention was measured using the same items as in Study 1. The Cronbach’s alpha of purchase intention was 0.76.

#### Analysis and Results

We first used ANOVA to test the effect of organic appeals advertising and emotional appeals on purchase intention toward organic milk. The results indicate that consumers who watched organic appeals advertisement (*M*_organic_=4.246, *SD*=0.505, *N*=99) held higher purchase intention toward organic milk than those who watched non-organic appeals advertisement (*M*_non-organic_=2.750, *SD*=0.517, *N*=56; *F*=308.094, *df*_between_=1, *df*_within_=153, *df*_total_=154, *p*<0.001). Thus, hypothesis H1 was further confirmed.

The results also indicate that consumers who listened to a happy song (*M*_positive_=3.961, *SD*=0.815, *N*=94) held higher purchase intention toward organic milk than those who listened to a sad song (*M*_negative_=3.311, *SD*=0.841, *N*=61; *F*=26.48, *df*_between_=1, *df*_within_=153, *df*_total_=154, *p*<0.001).

**Figure 2:**
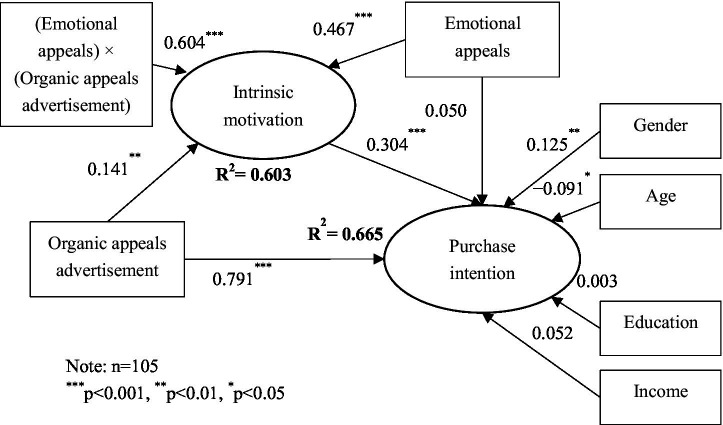
Results of hypothesis testing. *n*=105; ^***^p<0.001; ^**^p<0.01; ^*^p<0.05.

We further used structural equation modeling to test hypotheses H2 and H3. As indicated in Figure 2, some control variables were included in the model because of their potential effect. The results show that consumers’ gender (*β*=0.126, *p*<0.01) and age (*β*=−0.095, *p*<0.05) were significantly related to purchase intention. However, consumers’ education and income were not significantly related to purchase intention.

Results in Figure 2 show that organic appeals advertisement was significantly and positively related to intrinsic motivation (*β*=0.141, *p*<0.01), which in turn was significantly and positively related to purchase intention (*β*=0.304, *p*<0.001). To confirm the indirect effect of organic appeals advertisement on purchase intention through intrinsic motivation, we followed Preacher et al. (2007) and conducted a bootstrap analysis with 5,000 samples and a 95% confidence interval. The bootstrap analysis reveals that the indirect effect of organic appeals advertisement on purchase intention through intrinsic motivation was positively significant (organic appeals advertisement → intrinsic motivation → purchase intention: *β*=0.221, *p*<0.001, 95% CI=[0.076, 0.460]). Thus, hypothesis H2 was supported.

The results in Figure 2 also show that emotional appeals were positively related to intrinsic motivation (*β*=0.467, *p*<0.001) but not related to purchase intention (*β*=0.050, n.s.). Furthermore, the interaction effect between organic appeals advertisement and emotional appeals was significantly and positively associated with intrinsic motivation (*β*=0.604, *p*<0.001). To confirm this interaction effect, we performed a two-group analysis. The direct effect of organic appeals advertisement on intrinsic motivation was compared between positive and negative emotional appeals. The results indicate that organic appeals advertisement was positively related to intrinsic motivation for the positive emotional appeals group (*β*=0.357, *p*<0.001), but this relationship was not significant for the negative emotional appeals group (*β*=0.208, n.s.). Furthermore, the influence of organic appeals advertisement on intrinsic motivation was significantly different between positive and negative emotional appeals (∆*β*=0.149, *p*<0.01). Thus, hypothesis H3 was supported.

## Discussion and Conclusion

This study investigates the relationship between organic appeals advertising and purchase intention toward organic milk with the mediating role of intrinsic motivation and the moderating role of emotional appeals. The results show several interesting findings. Consumers receiving organic appeals advertising had greater purchase intention toward organic milk than those receiving non-organic appeals advertising. Furthermore, intrinsic motivation had a mediating effect on the relationship between organic appeals advertising and purchase intention. Furthermore, emotional appeals moderated the link between organic appeals advertising and intrinsic motivation.

### Theoretical Implications

First, although organic food has received great attention from researchers and business managers in the last few years, organic food sales volume remains low compared with conventional food (Sultan et al., 2020). In organic milk, the sales volume of conventional milk is much greater than organic milk. Although price is the main concern, the other reason that organic milk is less purchased is that consumers lack helpful information and knowledge about organic milk. Consumers do not know the benefits of organic milk and cannot distinguish between organic and non-organic milk products (Scozzafava et al., 2020). Given that few studies have determined the consumption of organic milk in prior food literature, the present study investigates antecedents of purchase intention toward organic milk. Thus, this study contributes to the current food literature by providing rich knowledge that explains consumer behavior toward organic milk.

Second, advertising is an important marketing tool for firms to communicate and persuade consumers to purchase products and services (Kotler and Armstrong, 2018). Surprisingly, how advertising influences consumer purchase behavior toward organic milk has been underdetermined in prior literature. This study based on SOR theory explained the relationship between organic appeals advertisement and purchase intention toward organic milk. Findings imply that organic appeals advertisement provides information and knowledge for consumers to distinguish between organic and conventional milk (Qin et al., 2009; Jaeger and Weber, 2020). Consumers who understand the benefits and superior quality of organic milk are more likely to purchase organic milk (Bloksma et al., 2008). Therefore, this study advances SOR theory and clarifies the relationship between organic appeals advertisement and purchase intention toward organic milk. Our findings provide implications for future researchers who may study the effect of organic appeals advertising on consumer behavior toward organic milk products.

Third, motivation often plays a vital role in affecting consumers’ attitudes and behavior in purchasing decisions (Soroka and Wojciechowska-Solis, 2019). Consumers may receive a signal from the external environment, which triggers their motivation to purchase a specific product (Standage et al., 2006). In the case of organic milk, following SDT logic (Ryan and Deci, 2000), we found that organic appeals advertisement enhances consumers’ intrinsic motivation, which increases their purchase intention toward organic milk. This finding indicates that organic appeals advertisement provides information and knowledge for consumers (Suciu et al., 2019). When consumers understand the benefits of organic milk, they are internally motivated toward organic milk.

Consequently, consumers may hold high intentions to purchase organic milk (Kushwah et al., 2019). Thus, the findings of this study extend SDT theory and shed new light on the mediating mechanism of intrinsic motivation, which has been absent in prior literature. Our findings provide implications for future researchers who may study the influence of motivation on purchase behavior toward organic milk products.

Finally, emotion is an important factor in consumers’ decision-making (Lajante and Ladhari, 2019). In this study, emotional appeals were found to have a moderating effect on the link between organic appeals advertisement and consumers’ purchase behavior toward organic milk. This finding implies that consumers who experience negative emotions often have negative feelings and unpleasant psychological states, resulting in negative attitudes and behavior (Xu, 2020). That is, negative emotion discourages consumers from receiving information and knowledge from organic appeals advertising. They also have low motivation toward organic milk products because negative emotion exerts psychological distress and uncomfortable feelings on consumers (Harrison-Walker, 2019). By contrast, positive emotion makes consumers pleasant and happy. In this case, consumers are strongly motivated to receive information and knowledge from organic appeals advertisements and tend to take purchasing behavior toward organic milk (Septianto and Chiew, 2018). Therefore, the findings of this study provide rich knowledge and clarify the moderating mechanism of emotional appeals, which has been unexplored in prior literature. Our results offer implications for future researchers who may study the role of emotion in affecting consumer decision-making toward organic milk products.

### Managerial Implications

Several implications are also provided for business managers in this study. It is suggested that business managers should plan and implement organic appeals advertising strategy to enhance consumers’ purchase behavior toward organic milk. Firms should provide detailed information in such advertisements and help consumers obtain helpful knowledge about organic milk products. Information in advertisements should also help consumers understand the benefits and distinguish between organic milk and traditional milk products. Furthermore, business managers should also have different strategies to trigger and increase consumers’ intrinsic motivation toward organic milk. For example, they may use a marketing campaign to persuade consumers that shopping for organic milk is a hedonic and pleasant process. This shopping behavior toward organic milk is internally necessary for consumers because consumption of organic milk brings health for consumers. Moreover, business managers should also have strategies to boost consumers’ positive emotions. For example, using emotional appeals (e.g., happy and suitable music, color, and store atmosphere) can elicit consumers’ positive feelings. As our experiment suggests, business managers should recruit experts to select the best emotional appeals to trigger consumers’ positive emotions.

### Limitations and Future Research

This study has several limitations that need to be addressed in future research. First, we conducted the experiment in Study 2, in which participants first saw the advertising and then listened to the songs. This sequence may generate a problem that the effect of the advertisement had faded away. However, if participants are exposed to the advertisement and the songs simultaneously, a compound effect between advertisement and songs may be another problem. In this case, we cannot distinguish the different effects between advertisement and emotional appeals on consumers’ intrinsic motivation and purchase intention. Thus, future research should adopt a more effective way to manipulate the independent effect (advertising) and moderating effect (emotional appeals) in this study. Second, although the experiment uses a good research design that can accurately test the causal relationship between variables, cross-sectional data of the experiment in this study may affect the accuracy of the causal relationship between variables. Thus, future research should use longitudinal data of experiments to validate the research model in this study. Third, we used two different advertisements (organic vs. non-organic) to manipulate organic appeals advertisement and two different songs (happy vs. sad songs) to manipulate emotional appeals. Although these manipulations were valid, future research should use different methods to conduct a better experiment. Finally, this study measured only the behavioral intention of consumers toward organic milk. Future research should measure real purchasing behavior better to observe consumers’ purchasing decisions toward organic milk.

## Data Availability Statement

The raw data supporting the conclusions of this article will be made available by the authors, without undue reservation.

## Ethics Statement

The studies involving human participants were reviewed and approved by the Major Project of The National Social Science Fund of China. Written informed consent for participation was not required for this study in accordance with the national legislation and the institutional requirements.

## Author Contributions

JW and MZ: conceptualization and supervision. JW, MZ, HW, W-D, and QW: methodology, formal analysis, and investigation. JW, MZ, and HW: original draft preparation and review and editing. All authors contributed to the article and approved the submitted version.

## Funding

Major Project of The National Social Science Fund of China (20ZDA087).

## Conflict of Interest

The authors declare that the research was conducted in the absence of any commercial or financial relationships that could be construed as a potential conflict of interest.

## Publisher’s Note

All claims expressed in this article are solely those of the authors and do not necessarily represent those of their affiliated organizations, or those of the publisher, the editors and the reviewers. Any product that may be evaluated in this article, or claim that may be made by its manufacturer, is not guaranteed or endorsed by the publisher.
